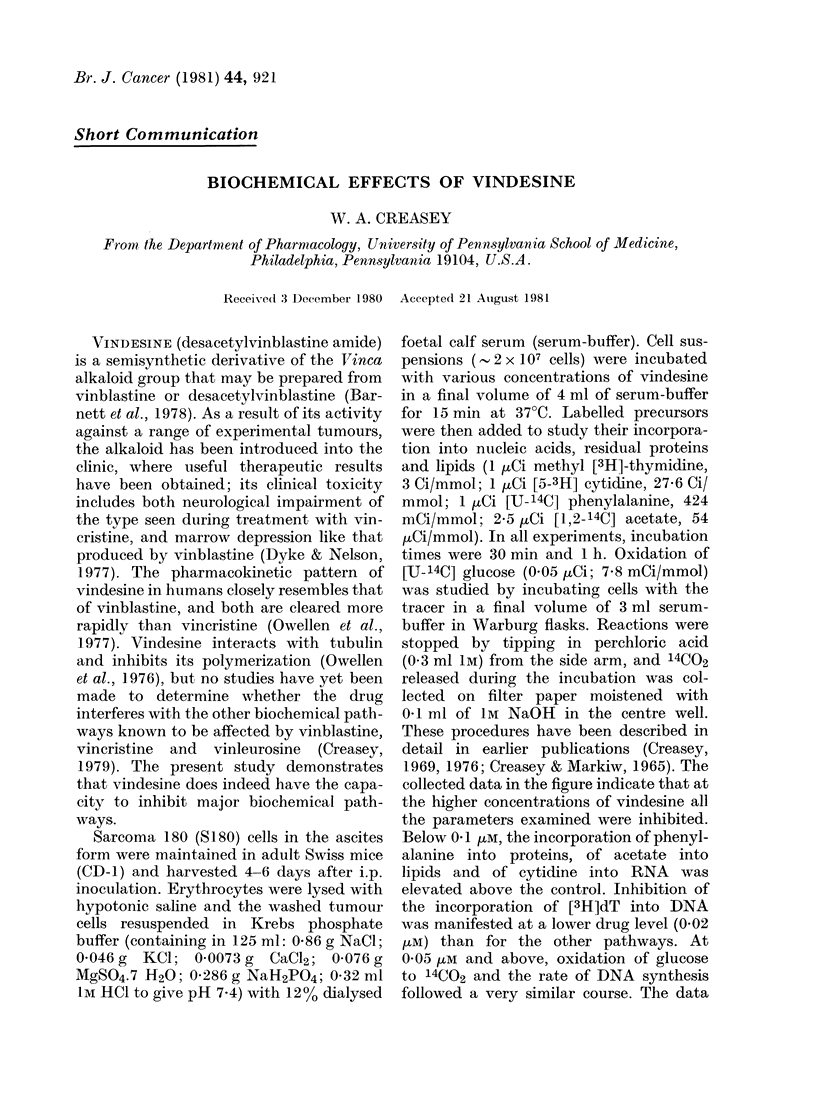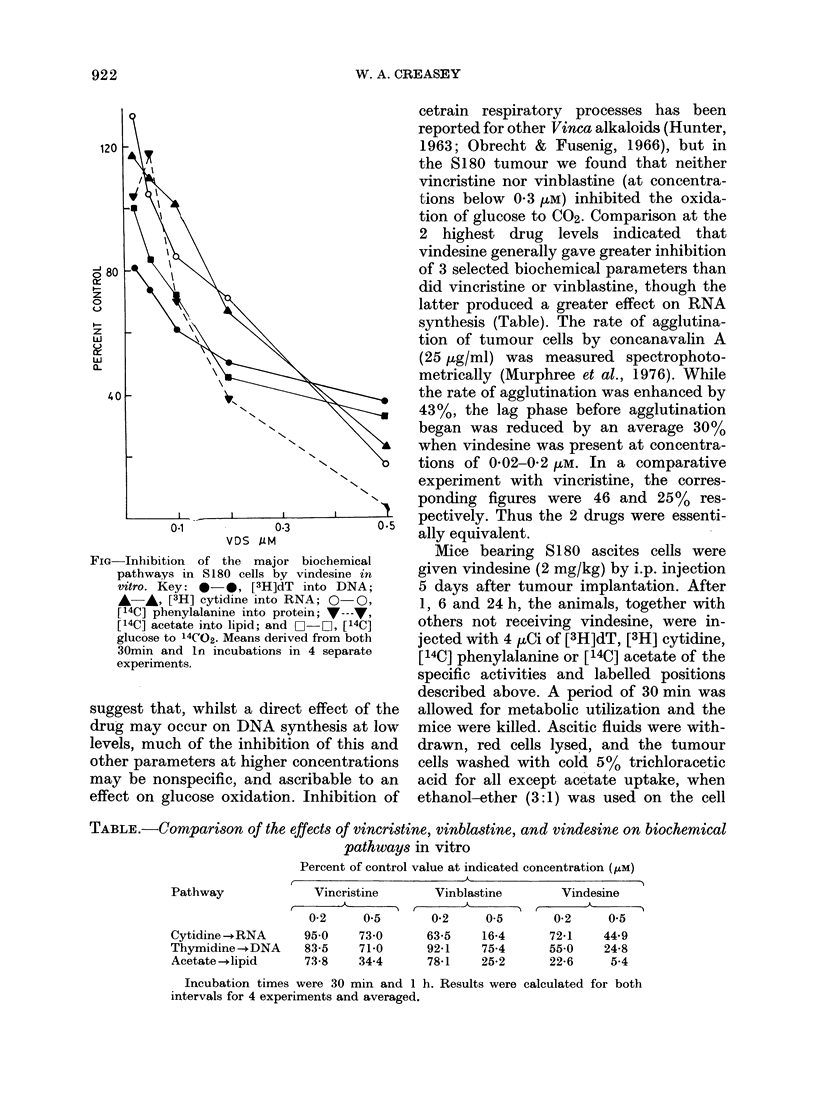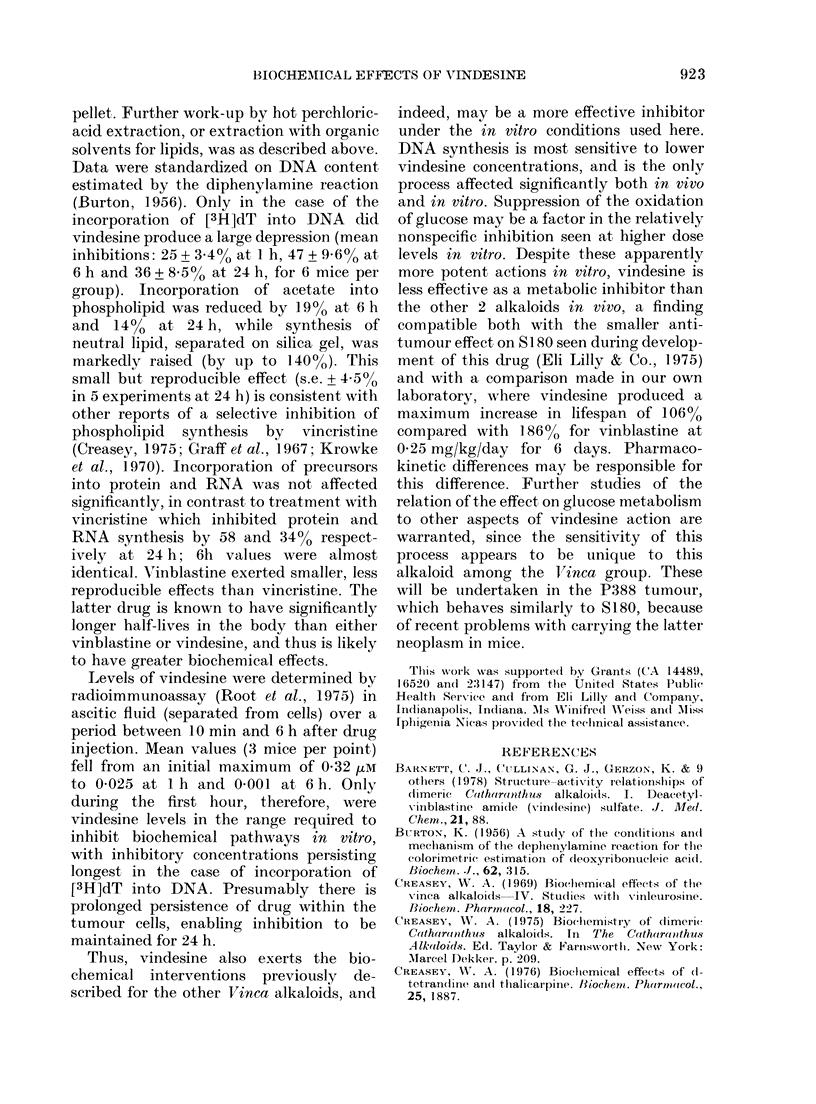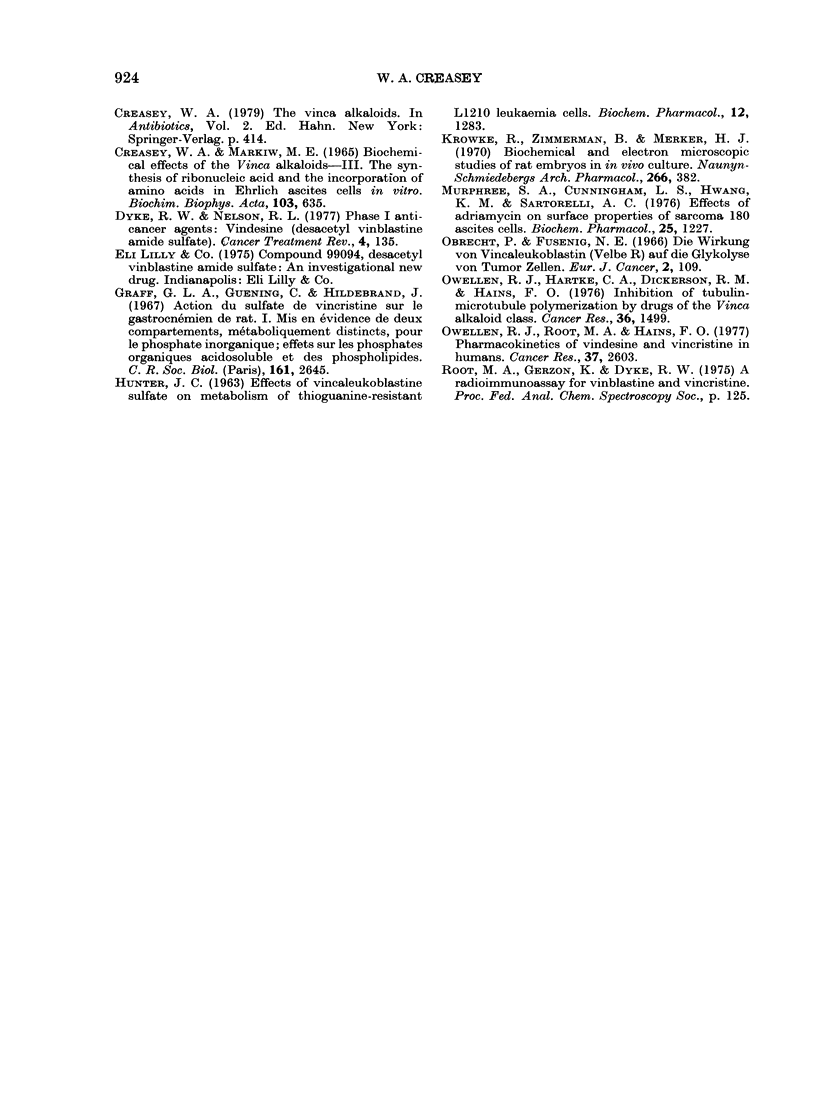# Biochemical effects of vindesine.

**DOI:** 10.1038/bjc.1981.293

**Published:** 1981-12

**Authors:** W. A. Creasey


*Br. J. Cancer* (1981) **44,** 921

***Short Communication***

### BIOCHEMICAL EFFECTS OF VINDESINE

W. A. CREASEY

*From the Department of Pharmacology, University of Pennsylvania School of Medicine, Philadelphia, Pennsylvania* 19104, *U.S.A.*

Received 3 December 1980 Accepted 21 August 1981

VINDESINE (desacetylvinblastine amide) is a semisynthetic derivative of the *Vinca* alkaloid group that may be prepared from vinblastine or desacetylvinblastine (Barnett *et al.*, 1978). As a result of its activity against a range of experimental tumours, the alkaloid has been introduced into the clinic, where useful therapeutic results have been obtained; its clinical toxicity includes both neurological impairment of the type seen during treatment with vincristine, and marrow depression like that produced by vinblastine (Dyke & Nelson, 1977). The pharmacokinetic pattern of vindesine in humans closely resembles that of vinblastine, and both are cleared more rapidly than vincristine (Owellen *et al.*, 1977). Vindesine interacts with tubulin and inhibits its polymerization (Owellen *et al.*, 1976), but no studies have yet been made to determine whether the drug interferes with the other biochemical pathways known to be affected by vinblastine, vincristine and vinleurosine (Creasey, 1979). The present study demonstrates that vindesine does indeed have the capacity to inhibit major biochemical pathways.

Sarcoma 180 (S180) cells in the ascites form were maintained in adult Swiss mice (CD-1) and harvested 4–6 days after i.p. inoculation. Erythrocytes were lysed with hypotonic saline and the washed tumour cells resuspended in Krebs phosphate buffer (containing in 125 ml: 0·86 g NaCl; 0·046 g KCl; 0·0073 g $CaCl_2$; 0·076 g $MgSO_4.7\ H_2O$; 0·286 g $NaH_2PO_4$; 0·32 ml 1M HCl to give pH 7·4) with 12% dialysed foetal calf serum (serum-buffer). Cell suspensions ($\sim 2 \times 10^7$ cells) were incubated with various concentrations of vindesine in a final volume of 4 ml of serum-buffer for 15 min at 37°C. Labelled precursors were then added to study their incorporation into nucleic acids, residual proteins and lipids (1 μCi methyl [$^3$H]-thymidine, 3 Ci/mmol; 1 μCi [5-$^3$H] cytidine, 27·6 Ci/mmol; 1 μCi [U-$^{14}$C] phenylalanine, 424 mCi/mmol; 2·5 μCi [1,2-$^{14}$C] acetate, 54 μCi/mmol). In all experiments, incubation times were 30 min and 1 h. Oxidation of [U-$^{14}$C] glucose (0·05 μCi; 7·8 mCi/mmol) was studied by incubating cells with the tracer in a final volume of 3 ml serum-buffer in Warburg flasks. Reactions were stopped by tipping in perchloric acid (0·3 ml 1M) from the side arm, and $^{14}CO_2$ released during the incubation was collected on filter paper moistened with 0·1 ml of 1M NaOH in the centre well. These procedures have been described in detail in earlier publications (Creasey, 1969, 1976; Creasey & Markiw, 1965). The collected data in the figure indicate that at the higher concentrations of vindesine all the parameters examined were inhibited. Below 0·1 μM, the incorporation of phenylalanine into proteins, of acetate into lipids and of cytidine into RNA was elevated above the control. Inhibition of the incorporation of [$^3$H]dT into DNA was manifested at a lower drug level (0·02 μM) than for the other pathways. At 0·05 μM and above, oxidation of glucose to $^{14}CO_2$ and the rate of DNA synthesis followed a very similar course. The data

FIG—Inhibition of the major biochemical pathways in S180 cells by vindesine *in vitro*. Key: ●—●, [$^3$H]dT into DNA; ▲—▲, [$^3$H] cytidine into RNA; ○—○, [$^{14}$C] phenylalanine into protein; ▼---▼, [$^{14}$C] acetate into lipid; and □—□, [$^{14}$C] glucose to $^{14}CO_2$. Means derived from both 30min and 1h incubations in 4 separate experiments.

suggest that, whilst a direct effect of the drug may occur on DNA synthesis at low levels, much of the inhibition of this and other parameters at higher concentrations may be nonspecific, and ascribable to an effect on glucose oxidation. Inhibition of cetrain respiratory processes has been reported for other *Vinca* alkaloids (Hunter, 1963; Obrecht & Fusenig, 1966), but in the S180 tumour we found that neither vincristine nor vinblastine (at concentrations below 0·3 μM) inhibited the oxidation of glucose to $CO_2$. Comparison at the 2 highest drug levels indicated that vindesine generally gave greater inhibition of 3 selected biochemical parameters than did vincristine or vinblastine, though the latter produced a greater effect on RNA synthesis (Table). The rate of agglutination of tumour cells by concanavalin A (25 μg/ml) was measured spectrophotometrically (Murphree *et al.*, 1976). While the rate of agglutination was enhanced by 43%, the lag phase before agglutination began was reduced by an average 30% when vindesine was present at concentrations of 0·02–0·2 μM. In a comparative experiment with vincristine, the corresponding figures were 46 and 25% respectively. Thus the 2 drugs were essentially equivalent.

Mice bearing S180 ascites cells were given vindesine (2 mg/kg) by i.p. injection 5 days after tumour implantation. After 1, 6 and 24 h, the animals, together with others not receiving vindesine, were injected with 4 μCi of [$^3$H]dT, [$^3$H] cytidine, [$^{14}$C] phenylalanine or [$^{14}$C] acetate of the specific activities and labelled positions described above. A period of 30 min was allowed for metabolic utilization and the mice were killed. Ascitic fluids were withdrawn, red cells lysed, and the tumour cells washed with cold 5% trichloracetic acid for all except acetate uptake, when ethanol–ether (3:1) was used on the cell

TABLE.—*Comparison of the effects of vincristine, vinblastine, and vindesine on biochemical pathways* in vitro

| Pathway | Percent of control value at indicated concentration (μM) | | | | | |
|---|---|---|---|---|---|---|
| | Vincristine | | Vinblastine | | Vindesine | |
| | 0·2 | 0·5 | 0·2 | 0·5 | 0·2 | 0·5 |
| Cytidine→RNA | 95·0 | 73·0 | 63·5 | 16·4 | 72·1 | 44·9 |
| Thymidine→DNA | 83·5 | 71·0 | 92·1 | 75·4 | 55·0 | 24·8 |
| Acetate→lipid | 73·8 | 34·4 | 78·1 | 25·2 | 22·6 | 5·4 |

Incubation times were 30 min and 1 h. Results were calculated for both intervals for 4 experiments and averaged.

pellet. Further work-up by hot perchloric-acid extraction, or extraction with organic solvents for lipids, was as described above. Data were standardized on DNA content estimated by the diphenylamine reaction (Burton, 1956). Only in the case of the incorporation of [$^3$H]dT into DNA did vindesine produce a large depression (mean inhibitions: $25 \pm 3{\cdot}4\%$ at 1 h, $47 \pm 9{\cdot}6\%$ at 6 h and $36 \pm 8{\cdot}5\%$ at 24 h, for 6 mice per group). Incorporation of acetate into phospholipid was reduced by 19% at 6 h and 14% at 24 h, while synthesis of neutral lipid, separated on silica gel, was markedly raised (by up to 140%). This small but reproducible effect (s.e. $\pm 4{\cdot}5\%$ in 5 experiments at 24 h) is consistent with other reports of a selective inhibition of phospholipid synthesis by vincristine (Creasey, 1975; Graff *et al.*, 1967; Krowke *et al.*, 1970). Incorporation of precursors into protein and RNA was not affected significantly, in contrast to treatment with vincristine which inhibited protein and RNA synthesis by 58 and 34% respectively at 24 h; 6h values were almost identical. Vinblastine exerted smaller, less reproducible effects than vincristine. The latter drug is known to have significantly longer half-lives in the body than either vinblastine or vindesine, and thus is likely to have greater biochemical effects.

Levels of vindesine were determined by radioimmunoassay (Root *et al.*, 1975) in ascitic fluid (separated from cells) over a period between 10 min and 6 h after drug injection. Mean values (3 mice per point) fell from an initial maximum of 0·32 $\mu$M to 0·025 at 1 h and 0·001 at 6 h. Only during the first hour, therefore, were vindesine levels in the range required to inhibit biochemical pathways *in vitro*, with inhibitory concentrations persisting longest in the case of incorporation of [$^3$H]dT into DNA. Presumably there is prolonged persistence of drug within the tumour cells, enabling inhibition to be maintained for 24 h.

Thus, vindesine also exerts the biochemical interventions previously described for the other *Vinca* alkaloids, and indeed, may be a more effective inhibitor under the *in vitro* conditions used here. DNA synthesis is most sensitive to lower vindesine concentrations, and is the only process affected significantly both *in vivo* and *in vitro*. Suppression of the oxidation of glucose may be a factor in the relatively nonspecific inhibition seen at higher dose levels *in vitro*. Despite these apparently more potent actions *in vitro*, vindesine is less effective as a metabolic inhibitor than the other 2 alkaloids *in vivo*, a finding compatible both with the smaller antitumour effect on S180 seen during development of this drug (Eli Lilly & Co., 1975) and with a comparison made in our own laboratory, where vindesine produced a maximum increase in lifespan of 106% compared with 186% for vinblastine at 0·25 mg/kg/day for 6 days. Pharmacokinetic differences may be responsible for this difference. Further studies of the relation of the effect on glucose metabolism to other aspects of vindesine action are warranted, since the sensitivity of this process appears to be unique to this alkaloid among the *Vinca* group. These will be undertaken in the P388 tumour, which behaves similarly to S180, because of recent problems with carrying the latter neoplasm in mice.

This work was supported by Grants (CA 14489, 16520 and 23147) from the United States Public Health Service and from Eli Lilly and Company, Indianapolis, Indiana. Ms Winifred Weiss and Miss Iphigenia Nicas provided the technical assistance.

### REFERENCES

Barnett, C. J., Cullinan, G. J., Gerzon, K. & 9 others (1978) Structure-activity relationships of dimeric *Catharanthus* alkaloids. I. Deacetylvinblastine amide (vindesine) sulfate. *J. Med. Chem.*, **21**, 88.

Burton, K. (1956) A study of the conditions and mechanism of the dephenylamine reaction for the colorimetric estimation of deoxyribonucleic acid. *Biochem. J.*, **62**, 315.

Creasey, W. A. (1969) Biochemical effects of the vinca alkaloids—IV. Studies with vinleurosine. *Biochem. Pharmacol.*, **18**, 227.

Creasey, W. A. (1975) Biochemistry of dimeric *Catharanthus* alkaloids. In *The Catharanthus Alkaloids*. Ed. Taylor & Farnsworth. New York: Marcel Dekker. p. 209.

Creasey, W. A. (1976) Biochemical effects of d-tetrandine and thalicarpine. *Biochem. Pharmacol.*, **25**, 1887.

CREASEY, W. A. (1979) The vinca alkaloids. In *Antibiotics*, Vol. 2. Ed. Hahn. New York: Springer-Verlag. p. 414.

CREASEY, W. A. & MARKIW, M. E. (1965) Biochemical effects of the *Vinca* alkaloids—III. The synthesis of ribonucleic acid and the incorporation of amino acids in Ehrlich ascites cells *in vitro*. *Biochim. Biophys. Acta*, **103**, 635.

DYKE, R. W. & NELSON, R. L. (1977) Phase I anticancer agents: Vindesine (desacetyl vinblastine amide sulfate). *Cancer Treatment Rev.*, **4**, 135.

ELI LILLY & CO. (1975) Compound 99094, desacetyl vinblastine amide sulfate: An investigational new drug. Indianapolis: Eli Lilly & Co.

GRAFF, G. L. A., GUENING, C. & HILDEBRAND, J. (1967) Action du sulfate de vincristine sur le gastrocnémien de rat. I. Mis en évidence de deux compartements, métaboliquement distincts, pour le phosphate inorganique; effets sur les phosphates organiques acidosoluble et des phospholipides. *C. R. Soc. Biol.* (Paris), **161**, 2645.

HUNTER, J. C. (1963) Effects of vincaleukoblastine sulfate on metabolism of thioguanine-resistant L1210 leukaemia cells. *Biochem. Pharmacol.*, **12**, 1283.

KROWKE, R., ZIMMERMAN, B. & MERKER, H. J. (1970) Biochemical and electron microscopic studies of rat embryos in *in vivo* culture. *Naunyn-Schmiedebergs Arch. Pharmacol.*, **266**, 382.

MURPHREE, S. A., CUNNINGHAM, L. S., HWANG, K. M. & SARTORELLI, A. C. (1976) Effects of adriamycin on surface properties of sarcoma 180 ascites cells. *Biochem. Pharmacol.*, **25**, 1227.

OBRECHT, P. & FUSENIG, N. E. (1966) Die Wirkung von Vincaleukoblastin (Velbe R) auf die Glykolyse von Tumor Zellen. *Eur. J. Cancer*, **2**, 109.

OWELLEN, R. J., HARTKE, C. A., DICKERSON, R. M. & HAINS, F. O. (1976) Inhibition of tubulin-microtubule polymerization by drugs of the *Vinca* alkaloid class. *Cancer Res.*, **36**, 1499.

OWELLEN, R. J., ROOT, M. A. & HAINS, F. O. (1977) Pharmacokinetics of vindesine and vincristine in humans. *Cancer Res.*, **37**, 2603.

ROOT, M. A., GERZON, K. & DYKE, R. W. (1975) A radioimmunoassay for vinblastine and vincristine. *Proc. Fed. Anal. Chem. Spectroscopy Soc.*, p. 125.